# Nephrin missense mutations: induction of endoplasmic reticulum stress and cell surface rescue by reduction in chaperone interactions

**DOI:** 10.1002/phy2.86

**Published:** 2013-09-17

**Authors:** Tetyana Drozdova, Joan Papillon, Andrey V Cybulsky

**Affiliations:** Department of Medicine, McGill University Health Centre, McGill UniversityMontreal, Quebec, Canada

**Keywords:** Calnexin, glomerulonephritis, proteinuria, unfolded protein response

## Abstract

Nephrin, an important component of the podocyte filtration slit diaphragm, plays a key role in the maintenance of glomerular permselectivity. Mutations in nephrin lead to proteinuria and congenital nephrotic syndrome. Nephrin undergoes posttranslational modifications in the endoplasmic reticulum (ER) prior to export to the plasma membrane. We examined the effects of human nephrin disease-associated missense mutations on nephrin folding in the ER and on cellular trafficking in cultured cells. Compared with wild-type (WT) nephrin, the mutants showed impaired glycosylation and enhanced association with the ER chaperone, calnexin, as well as accumulation in the ER. Nephrin mutants demonstrated enhanced ubiquitination, and they underwent ER-associated degradation. Certain nephrin mutants did not traffic to the plasma membrane. Expression of nephrin mutants resulted in the stimulation of the activating transcription factor-6 pathway of the unfolded protein response, and an increase in the ER chaperone, Grp94. We treated cells with castanospermine (an inhibitor of glucosidase I) in order to decrease the association of nephrin mutants with calnexin. Castanospermine increased plasma membrane expression of nephrin mutants; however, full glycosylation and signaling activity of the mutants were not restored. Modulation of ER quality control mechanisms represents a potential new approach to development of therapies for proteinuric kidney disease, including congenital nephrotic syndrome.

## Introduction

The glomerular visceral epithelial cell (GEC; podocyte) is a key component of the glomerular capillary wall and plays an important role in the maintenance of glomerular permselectivity (Tryggvason et al. 2006). Nephrin is located in podocyte foot processes, and is the principal component of filtration slit diaphragms (Ruotsalainen et al. 1999; Tryggvason et al. 2006; Patrakka and Tryggvason 2007). Nephrin was discovered through positional cloning, which identified the NPHS1 gene as the cause of congenital nephrotic syndrome of the Finnish type (Kestila et al. 1998). This discovery not only provided an understanding of the pathophysiology of this disease but also novel insights into the functioning of the glomerular filtration barrier. Nephrin is a transmembrane glycoprotein belonging to the immunoglobulin superfamily (Tryggvason et al. 2006; Patrakka and Tryggvason 2007). It consists of eight extracellular domains, followed by a fibronectin type III–like domain, a short transmembrane region, and a short cytoplasmic C-terminus. Based on the primary structure, it could be predicted that nephrin molecules from adjacent foot processes interact in the center of the podocyte filtration slit diaphragm to form a zipper-like structure, and filtration channels, too small for albumin-sized molecules to pass (Tryggvason et al. 2006). More recent work indicates that nephrin homotypic interactions may influence cytoplasmic posttranslational modifications and signaling (Tryggvason et al. 2006; Chuang and He 2009; Hattori et al. 2011).

The endoplasmic reticulum (ER) is an organelle where secretory and membrane proteins, such as nephrin, are processed after translation. Nascent proteins are folded in the ER with the assistance of molecular chaperones and folding enzymes, and only correctly folded proteins are transported to the Golgi apparatus (Araki and Nagata 2011). To rescue misfolded proteins, the ER has in place quality control systems that allow aberrant proteins multiple opportunities to acquire their correct conformation (Araki and Nagata 2011; Brodsky and Skach 2011). ER quality control machinery encompasses two major groups of proteins, including the molecular chaperones and glycan modification enzymes. Classical chaperones such as BiP (Grp78) and Grp94 are involved in the general folding process of secretory proteins. Calnexin and calreticulin are lectin chaperones, specifically involved in the folding of glycoproteins (Deprez et al. 2005; Caramelo and Parodi 2008; Araki and Nagata 2011; Brodsky and Skach 2011). High-mannose–type oligosaccharide is attached en bloc to most proteins translocated into the ER, and is then trimmed sequentially. When two glucose residues are trimmed by glucosidase I or II, and the protein contains only one glucose residue, calnexin or calreticulin binds and folds the client protein. After the last glucose residue is trimmed by glucosidase II, the client protein is released from calnexin/calreticulin. Ultimately, the folded protein is transported to the Golgi apparatus for additional modification. If a protein is folded incorrectly, the protein may be reglucosylated, thereby allowing it to rebind to calnexin (the “calnexin cycle”) (Deprez et al. 2005; Caramelo and Parodi 2008; Araki and Nagata 2011; Brodsky and Skach 2011). Proteins that remain misfolded in the ER despite repeated attempts at folding undergo ER-associated degradation (ERAD) (Hirsch et al. 2009; Araki and Nagata 2011; Bernasconi and Molinari 2011; Guerriero and Brodsky 2012). In ERAD, proteins interact with a specific set of chaperones, are retrotranslocated to the cytosol, and are ubiquitinated and degraded by the proteasome. Some misfolded proteins tend to form aggregates, and these aggregates could potentially inhibit the ubiquitin-proteasome system by saturating the capacity of one or more molecular chaperones, or impairing components of ubiquitin-proteasome function (Bence et al. 2001; Dantuma and Lindsten 2010).

When the amount of misfolded protein in the ER exceeds the capacity of the folding apparatus and ERAD machinery, the misfolded proteins lead to ER stress and activation of the unfolded protein response (UPR) (Ron and Walter 2007; Yoshida 2007; Zhang and Kaufman 2008; Hetz 2012). Three major signaling pathways comprise the UPR. These are initiated by the protein sensors, activating transcription factor-6 (ATF6), inositol-requiring-1α, and PERK (PKR-like ER kinase). In resting cells, BiP binds to the luminal domains of UPR sensors and keeps them from being activated. Misfolded proteins can activate the UPR sensors via competition with BiP, or via direct binding to the sensors (Yoshida 2007). Upon accumulation of misfolded proteins, ATF6 is released from BiP and moves to the Golgi, where it is cleaved by site-1 and site-2 proteases. The cleaved cytosolic fragment migrates to the nucleus to activate transcription of ER chaperones and ERAD components, enhancing the capacity of the cell for protein folding. In parallel, inositol-requiring 1α autophosphorylates and activates its endoribonuclease activity, cleaving X-box-binding protein-1 mRNA and changing the reading frame to yield a potent transcriptional activator of the aforementioned genes. A third aspect of the UPR involves PERK, which is activated through homodimerization and transphosphorylation. PERK phosphorylates the eukaryotic translation initiation factor-2α subunit (eIF2α), leading to translational attenuation, thereby reducing the protein load on a damaged ER. When ER stress is intense, phosphorylation of eIF2α can also upregulate the expression of ATF4, leading to enhanced transcription of proapoptotic target genes, including CHOP (C/EBP homologous protein 10 or growth arrest and DNA damage 153).

Nephrin is normally glycosylated and folded in the ER before transport to the plasma membrane (Yan et al. 2002). Human nephrin has 10 potential N-glycosylation sites in its amino acid sequence, and N-glycosylation has been demonstrated at nine sites (Khoshnoodi et al. 2007; Patrakka and Tryggvason 2007). When cells were treated with the N-linked glycosylation inhibitor, tunicamycin, plasma membrane trafficking of nephrin was blocked and underglycosylated nephrin remained in the ER, suggesting a critical role of the N-linked glycosylations in nephrin membrane trafficking (Yan et al. 2002). More than 90 different mutations have been identified in Finnish and non-Finnish patients with congenital nephrotic syndrome (Lenkkeri et al. 1999; Beltcheva et al. 2001; Koziell et al. 2002), the most common (more than 60) being missense mutations. The latter typically result in the synthesis of a fully mature protein, but with amino acid substitutions, which may induce unfavorable conformations, or incorrect disulfide bridges (Liu et al. 2001). Nephrin missense mutations are scattered along the entire polypeptide, and often cause the same severe phenotype as deletion or insertion mutations (Liu et al. 2001). It was found that many of the nephrin missense mutants were not transported to the cell surface, and some were shown to accumulate in the ER (Liu et al. 2001).

Protein misfolding and stress pathway activation in the ER are emerging as central to the impaired glomerular permselectivity in experimental glomerular diseases, particularly diseases of the podocyte (Cybulsky 2010). In the ER, the UPR and ERAD surveillance must strike a fine balance protecting podocytes from a proteotoxic effects of misfolded proteins, while avoiding disposal of mildly affected proteins, particularly those that are important to glomerular permselectivity, including nephrin. Expression of nephrin in slit diaphragms is reduced in both congenital and acquired proteinuric diseases, but understanding of the mechanisms is limited (Patrakka and Tryggvason 2007). In this study, we demonstrate that nephrin mutants accumulate in the ER, show enhanced association with calnexin, undergo ERAD, and lead to the induction of the ATF6 branch of the UPR. Certain nephrin mutants are poorly expressed in the plasma membrane, and plasma membrane expression could be enhanced by reducing the interaction of the nephrin mutant with calnexin.

## Material and Methods

### Materials

Tissue culture reagents and the Lipofectamine 2000 transfection reagent were obtained from Invitrogen (Burlington, ON). Electrophoresis and immunoblotting reagents were from Jackson ImmunoResearch (West Grove, PA), Fermentas Inc. (Burlington, ON), and Pall Corp. (Mississauga, ON). Tunicamycin was obtained from Sigma-Aldrich Canada (Mississauga, ON). Rabbit antinephrin antibody (which reacts with the cytoplasmic domain of nephrin) was kindly provided by Dr. Tomoko Takano (McGill University) (Zhu et al. 2008). Rabbit antinephrin antibody (which reacts with the extracellular domain of nephrin; H-300), mouse antigreen fluorescent protein (GFP), and rabbit anti-CHOP antibodies were from Santa Cruz Biotechnology (Santa Cruz, CA). Rat anti-Grp94, mouse anticalnexin, and rabbit anticalreticulin antibodies were from Stressgen/Assay Designs (Ann Arbor, MI). Rabbit antiphospho-eIF2α was purchased from Invitrogen. Endoglycosidase H was from New England Biolabs (Mississauga, ON). Human wild-type (WT) nephrin cDNA was provided by Dr. Takano (Huber et al. 2001; Zhu et al. 2008). Nephrin mutant cDNAs, encoding S366R, G270C, R743C, S724C, and I171N, were kindly provided by Dr. Karl Tryggvason (Karolinska Institute, Stockholm, Sweden) (Liu et al. 2001). The plasmid 5xATF6-GL3 was purchased from Addgene (Cambridge, MA) (Wang et al. 2000). The AP1 firefly luciferase reporter plasmid was described previously (Huber et al. 2001).

### Cell culture

Most experiments were carried out in human embryonic kidney 293T cells, which do not express endogenous nephrin, and may be transfected with high efficiency. The 293T cells were cultured on plastic substratum in Dulbecco's modified eagle medium (DMEM) supplemented with 10% fetal bovine serum. All experiments were conducted between passages 20 and 50. Certain results were confirmed in rat GECs and COS-1 cells. GECs are a more physiological model, but their transfection efficiency is low. GECs were established from primary cultures, and were characterized previously, and were cultured in K1 medium on plastic substratum (Kitzler et al. 2012; Coers et al. 1996). Experiments were conducted between passages 30 and 55. COS-1 cells, which do not express endogenous nephrin, are derived from monkey kidney, and were cultured in DMEM with 10% fetal bovine serum. A clonal line of 293 cells stably expressing GFP^U^ (Bence et al. 2001) was purchased from American Type Culture Collection (Manassas, VA), and were cultured similar to the 293T cells.

### Transfection and cell harvesting

Cells were transiently transfected with plasmid DNAs, using the Lipofectamine 2000 reagent, according to the procedure provided by manufacturer (Kitzler et al. 2012). Cells were harvested in buffer containing 1% Triton X-100, 125 mmol/L NaCl, 10 mmol/L Tris (pH 7.5), 1 mmol/L ethylenediaminetetraacetic acid, 2 mmol/L Na_3_VO_4_, 5 mmol/L Na_4_P_3_O_7_, and 25 mmol/L NaF with protease inhibitor cocktail (Fermentas) 10 μL/mL. For study of ubiquitination, 10 mmol/L N-ethylmaleimide was included in the lysis buffer. Lysates were centrifuged for 10 min at 10,000*g*. Protein concentrations in supernatants were measured via the Bradford assay.

### Immunoprecipitation and immunoblotting

Cell lysates were incubated with agarose for 1 h at 4°C (preclearance). Supernatants were incubated overnight at 4°C with primary antibody, or nonimmune IgG in controls. After incubation with Protein A-agarose (Millipore, Temecula, CA) and washing, samples were boiled for 5 min in Laemmli buffer and subjected to sodium dodecyl sulfate polyacrylamide gel electrophoresis (SDS-PAGE). Proteins were then electrophoretically transferred to a nitrocellulose membrane. After blocking, membranes were incubated with primary antibody overnight at 4°C and then with secondary antibody at 22°C for 1 h. Western blots were developed by enhanced chemiluminescence. Density of specific bands was measured using National Institutes of Health Image J software. Preliminary studies demonstrated that there was a linear relationship between densitometric measurements and the amounts of protein loaded onto gels (Kitzler et al. 2012).

### Digestion with endoglycosidase H

Cells were scraped from culture plates, pelleted, and solubilized and boiled in 0.5% SDS, 40 mmol/L dithiothreitol for 10 min. Lysates (30 μg of protein) were digested with 750 units of endoglycosidase H in 0.05 mol/L sodium citrate, pH 5.5 (30 μL) for 2 or 18 h at 37°C. Then, samples were boiled in Laemmli buffer and subjected to SDS-PAGE.

### Dual luciferase reporter assay

Cells were transiently transfected with WT nephrin, or nephrin mutants, together with a firefly luciferase reporter cDNA and a cDNA-encoding renilla luciferase, using Lipofectamine 2000. Cells were cultured for 48 h. Lysates were assayed for firefly and renilla luciferase activities using a kit (Dual Luciferase Reporter Assay System, Promega, Madison, WI) according to manufacturer's specifications. Measurements were performed in a Lumat LB 9507 luminometer (Berthold, Oak Ridge, TN), and the ratios between firefly and renilla luciferase activities (the latter a marker of transfection efficiency) were calculated.

### Immunofluorescence microscopy

Cells plated on glass cover slips were transfected and cultured for 48 h. Then, cells were washed and fixed in 4% formaldehyde (in phosphate buffered saline [PBS]) for 15 min. In some experiments, the cells were permeabilized with 0.5% Triton X-100 in PBS for 3 min. After blocking (3% bovine serum albumin in PBS, 30 min), cells were incubated with primary antibodies for 30–60 min (antinephrin) or overnight (anticalnexin) at 22°C, followed by the appropriate secondary fluorophore-conjugated antibodies for 60 min at 22°C. Nuclei were counterstained with Hoechst H33342 dye (1 μg/mL in PBS) for 6 min at 22°C. Permeabilized cells were incubated with rhodamine phalloidin (0.04 μg/mL). Coverslips were mounted onto glass slides using medium containing an antifade component. Images were acquired at intensity below saturation using a Zeiss AxioObserver fluorescence microscope with visual output connected to an AxioCam digital camera (Zeiss, Toronto, ON). The images were collected from series of images derived from different focal planes (Z-stack).

Relative measurements of fluorescence intensity of nephrin at the cell surface were carried out with Adobe Photoshop software (Bijian et al. 2005; Kirkeby and Thomsen 2005). In these experiments, nonpermeable cells were stained with antinephrin antibody plus fluorescein-conjugated secondary. Then, cells were permeabilized and stained with rhodamine phalloidin. Fluorescein fluorescence intensity (reflecting nephrin on the cell surface) was measured by quantifying the average value of the green channel in the photographs of the cells using the Histogram tool of Adobe Photoshop, whereas rhodamine-phalloidin fluorescence (reflecting F-actin content) was measured by quantifying the average value of the red channel. In preliminary studies, we determined that rhodamine-phalloidin fluorescence was highly correlated with the area of the photograph occupied by cells (*R* = 0.92, *P* = 1 × 10^5^). Green fluorescence was then normalized for red fluorescence, and results were expressed in arbitrary units. In untransfected stained cells, fluorescein fluorescence intensity was undetectable.

### Statistics

Results are presented as mean ± SEM. One-way analysis of variance was used to determine significant differences among groups. Where significant differences were found, individual comparisons were made between groups using the t statistic, and adjusting the critical value according to the Bonferroni method.

## Results

### Nephrin mutants do not undergo complete oligosaccharide processing and show enhanced association with calnexin

**Figure 1:**
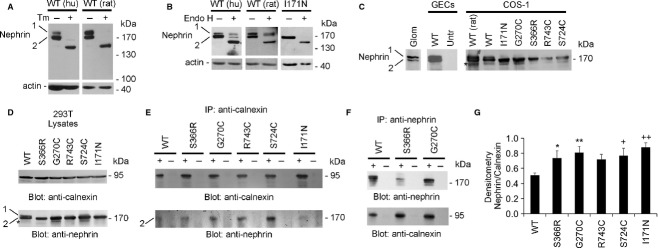
Human nephrin mutants do not undergo complete oligosaccharide processing and show enhanced association with calnexin. (A) 293T cells were transiently transfected with human (hu) or rat nephrin wild-type (WT) cDNAs. Cells were incubated with or without tunicamycin (Tm; 10 μg/mL) for 24 h. In lysates of untreated cells, nephrin migrated as two bands (∼180 kDa). Tunicamycin induced a loss of the doublet and the appearance of a single faster migrating band. (B) Lysates of 293T cells expressing human or rat WT nephrin, or human I171N mutant, were incubated with or without endoglycosidase (endo) H for 2 h. Endoglycosidase H induced a nearly complete loss of the lower nephrin band (band 2) and the appearance of a faster migrating band. Band 1 in WT was largely unaffected. Therefore, band 1 most likely represents the fully mature form of nephrin, carrying complex oligosaccharide, and band 2 is an immature, high-mannose form. (C and D) Expression of endogenous nephrin in isolated mouse glomeruli (Glom), and expression of human and rat nephrin WT, and human nephrin mutants in GECs, COS-1 cells, and 293T cells after transfection. Expression of endogenous nephrin is not detectable in GECs. In COS-1 and 293T cells, a faint third band marked by the asterisk is probably a degradation product. (E) The association of nephrin with calnexin was studied by immunoprecipitation (IP) with antibody to calnexin (+), or nonimmune IgG in controls (−), followed by immunoblotting of immune complexes with antinephrin antibody. The nephrin that binds to calnexin (i.e., WT and mutants; lower panel) is the partially glycosylated form (band 2). Compared with nephrin WT, most mutants showed significantly increased association with calnexin. The content of nephrin and calnexin in cell lysates is shown in panel D. (F) Association of nephrin with calnexin was also studied in WT and two mutants by immunoprecipitation (IP) with antibody to nephrin (+), or nonimmune IgG in controls (−), followed by immunoblotting of immune complexes with anticalnexin antibody. Compared with WT, the two mutants showed increased association of nephrin with calnexin. (G) Densitometric quantification of experiments in panel E, that is, immunoprecipitated nephrin, normalized for calnexin. *P < 0.045, **P < 0.03, ^+^*P* = 0.07, ^++^*P* < 0.006, mutant versus WT, *N* = 4 (R743C was not significant).

For this study, we selected five human nephrin mutants, which cause glomerular disease in humans (Liu et al. 2001). The I171N, G270C, and S366R mutations are found in the immunoglobulin-like domains of nephrin, and these mutations were reported to abolish cell surface expression of nephrin. The S366R mutant was previously shown to accumulate in the ER (Liu et al. 2001). The S724C and R743C mutations are in the spacer region of nephrin, and these mutants were reported to traffic to the cell surface (Liu et al. 2001). First, we examined the expression of nephrin in 293T cells, which do not express endogenous nephrin. By SDS-PAGE and immunoblotting, ectopic human and rat WT nephrin in 293T cells could be resolved into a doublet (Fig. 1A and B, bands 1 and 2), with the two bands migrating closely together at ∼180 kDa. Incubation of cells with tunicamycin, a drug that blocks all N-glycosylation of proteins, resulted in the loss of the doublet and the appearance of a single faster migrating band, most likely representing nonglycosylated nephrin (Fig. 1A). Endoglycosidase H is a specific endoglycosidase, which primarily cleaves asparagine-linked mannose-rich oligosaccharides, but not highly processed complex oligosaccharides from glycoproteins. Treatment of lysates of cells expressing human or rat WT nephrin with endoglycosidase H for 2 h resulted in an almost complete loss of the lower nephrin band (band 2; Fig. 1B) and the appearance of a faster migrating band, which was of the same molecular mass as the band induced by tunicamycin treatment. A similar result was observed with an 18 h incubation (not shown). Based on these experiments, it can be concluded that the upper band (band 1) represents the fully mature form of nephrin, carrying complex oligosaccharide (i.e., underwent complete oligosaccharide processing in the Golgi and trafficked to the cell surface) (Khoshnoodi et al. 2007), whereas band 2 is a high-mannose, immature form. These two forms of nephrin have also been referred to as “a” and “b” (Liu et al. 2001).

To confirm the results in 293T cells, we examined expression of nephrin WT in the glomerulus and two other cultured cell lines, which do not express endogenous nephrin (Fig. 1C and D). By analogy to 293T cells, GECs (a physiologically relevant model for nephrin expression) and COS-1 cells transfected with nephrin WT showed that the protein migrated as a doublet, representing the fully mature form with complex oligosaccharide, and the high-mannose form. For comparison, in isolated mouse glomeruli (Cybulsky et al. 2010), endogenous nephrin was also expressed as both fully glycosylated and partially glycosylated forms (Fig. 1C). In contrast, all the nephrin mutants expressed in 293T cells and COS-1 cells were expressed only as partially glycosylated forms (band 2; Fig. 1C and D, lower panel). In some experiments, a faint lower band was also visible (Fig. 1C and D, asterisk); this band was not present consistently, and most likely represents a degradation product. Treatment of lysates of cells expressing the human I171N nephrin mutant with endoglycosidase H resulted in a complete loss of the single nephrin band (band 2) and the appearance of the faster migrating band, representing nonglycosylated nephrin (Fig. 1B). This experiment confirms that nephrin mutants are expressed only as high-mannose, immature forms.

Calnexin is an ER chaperone, whose main function is to assist with protein folding and quality control, ensuring that only properly folded and assembled proteins proceed further along the secretory pathway. We hypothesized that the nephrin mutants are misfolded N-linked glycoproteins, and will show enhanced retention in the ER and binding to calnexin. The association of nephrin with calnexin was studied by immunoprecipitation with antibody to calnexin, followed by immunoblotting of the immune complexes with antinephrin antibody (Fig. 1D and E). All the nephrin mutants bound to calnexin immunoprecipitates, whereas only the high-mannose form of WT nephrin was detectable, that is, a nephrin WT doublet was not present in these immunoprecipitates (Fig. 1E). Compared with WT nephrin, the S366R, G270C, and I171N mutants showed significantly increased association with calnexin, suggesting that these mutants were severely misfolded, whereas the S724C and R743C mutants tended to show enhanced association, suggesting milder misfolding (Fig. 1E and G). An analogous experiment was performed with nephrin WT, and two of the more misfolded mutants (S366R and G270C), using immunoprecipitation with antibody to nephrin, followed by immunoblotting of the immune complexes with anticalnexin antibody (Fig. 1F). The S366R and G270C mutants showed increased association with calnexin, compared with WT nephrin. Therefore, the partially glycosylated form of nephrin (band 2) binds to calnexin, implying that this form is found in the ER.

### Nephrin mutants are localized in the ER

**Figure 2:**
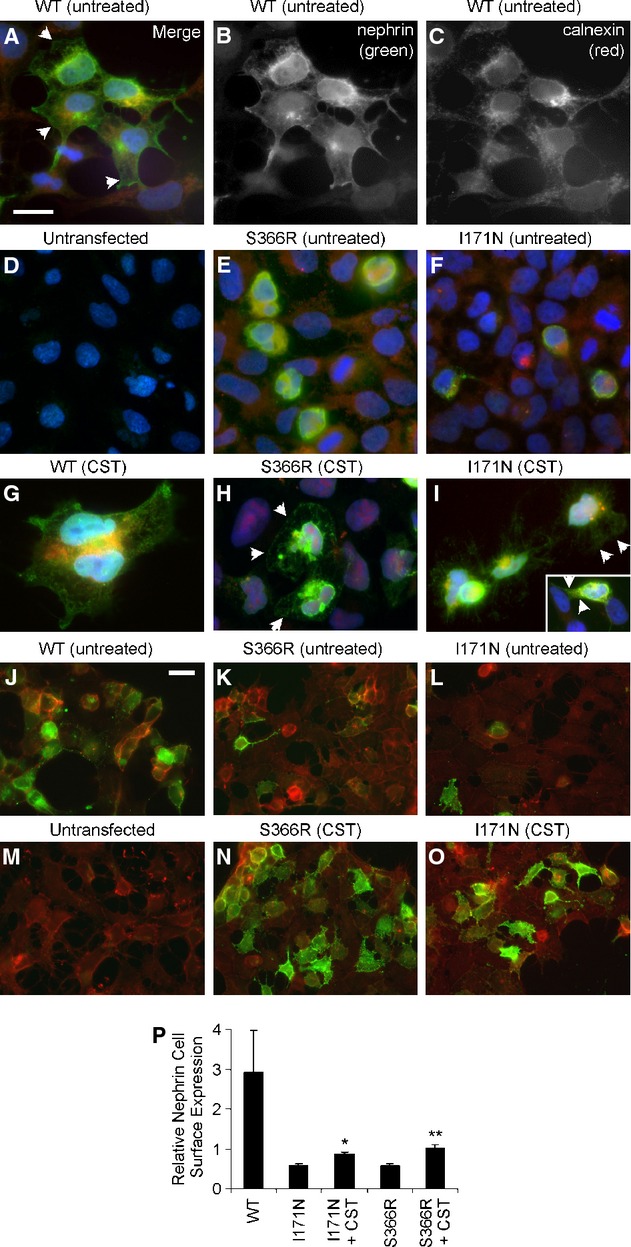
Nephrin expression and localization (immunofluorescence microscopy, Z-stack images). 293T cells were transfected with nephrin WT or the S366R and I171N mutants, as indicated. (A–F) After 48 h, cells were fixed, permeabilized and stained with antibody to the nephrin cytoplasmic domain (green) and antibody to calnexin (red). Nuclei were stained with Hoechst (blue). Calnexin staining (A and C) is generally perinuclear. Staining of nephrin WT (A and B) is in the plasma membrane (arrowheads), and there is also some perinuclear staining, which colocalizes with calnexin (A; yellow-orange staining). (D) Staining of untransfected cells with antibody to the nephrin cytoplasmic domain was negative. (E and F) In contrast to nephrin WT, the S366R and I171N nephrin mutants mainly showed perinuclear staining, and there was costaining with calnexin. (G–I) Cells were incubated with castanospermine (CST; 1 mmol/L, 18 h). Cells were fixed, permeabilized, and stained with antibodies as in A–F. Castanospermine had no significant effect on nephrin WT (G), but increased the amount of nephrin S366R and I171N appearing in the plasma membrane (arrows). (J–L, N–O, and P) At 48 h after transfection, nonpermeabilized cells were fixed and stained with antibody to the nephrin extracellular domain (green). Then, cells were permeabilized and incubated with rhodamine phalloidin (red) to visualize F-actin. Some cells (N and O) were first treated with castanospermine, as above. Nephrin WT is expressed on the cell surface (J), whereas surface expression of nephrin S366R and I171N is detected in only a few cells (K and L). Castanospermine increased expression of nephrin S366R and I171N on the cell surface (N and O). (M) Staining of untransfected cells with antibody to the nephrin extracellular domain was negative. Panels A–I and J–O are at similar magnifications; the bars = 15 μm. (P) Quantification of fluorescence intensity (green, which reflects nephrin plasma membrane expression, and red, reflecting total F-actin; see Materials). Nephrin expression was normalized for F-actin content. Castanospermine enhanced plasma membrane expression of the nephrin mutants. *P < 0.0002, **P < 0.0001 versus untreated, *N* = 15.

To verify the above results, we compared the subcellular localization of nephrin WT with the nephrin S366R and I171N mutants in 293T cells, using immunofluorescence microscopy. By immunostaining permeabilized cells with an antibody to the nephrin cytoplasmic domain, we demonstrated that nephrin WT was localized in the plasma membrane. There was also perinuclear staining that colocalized with the ER chaperone calnexin, which was used as a marker of the ER (Fig. 2A–C). A similar plasma membrane and perinuclear nephrin staining pattern were evident in GECs (result not shown). This result is consistent with the pattern of nephrin glycosylation (Fig. 1), that is, immunostaining at the plasma membrane most likely corresponds to the nephrin carrying complex oligosaccharide, whereas ER staining represents the high-mannose form. In contrast to nephrin WT, the S366R and I171N nephrin mutants in 293T cells showed staining that was principally aggregated in the cytoplasm in a perinuclear distribution (Fig. 2E and F). This staining was colocalized with calnexin, indicating that the S366R and I171N nephrin mutants were retained in the ER, and were not detected in the plasma membrane. By analogy, the G270C nephrin mutant was localized in the ER (result not shown). Immunostaining of nonpermeabilized 293T cells with an antibody to the extracellular domain of nephrin demonstrated an abundance of nephrin WT expressed in the plasma membrane (Fig. 2J). Using this technique, cell surface expression of nephrin S366R and I171N mutants could be detected in a small number of cells (Fig. 2K and L). These results confirm that the S366R and I171N mutations impair export to the plasma membrane significantly.

### Nephrin mutants undergo degradation

**Figure 3:**
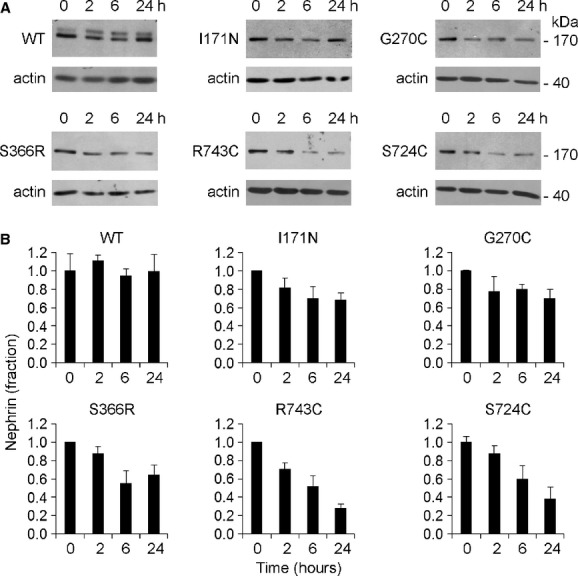
Nephrin mutants undergo ER-associated degradation (ERAD). 293T cells were transiently transfected with nephrin WT or mutants. After addition of cycloheximide (25 μmol/L; to block protein synthesis), nephrin expression was monitored by immunoblotting of cell lysates with antinephrin antibody at 0, 2, 6, and 24 h. Representative immunoblots (A) and densitometric quantification (B) are presented. As both nephrin WT bands appeared stable over 24 h, the two bands were quantified together. Expression declined in all mutants, consistent with ERAD.

Proteins that are terminally misfolded in the ER may undergo ERAD, which involves transport to the cytosol (retrotranslocation), ubiquitination, and degradation by the proteasome. Typically, such proteins are unstable and show a reduced half-life. In the next set of experiments, we examined the stability of nephrin proteins. 293T cells were transfected with nephrin WT and mutants, and after addition of cycloheximide (to block further protein synthesis), the expression of nephrin protein was monitored by immunoblotting at serial time intervals. Expression of nephrin WT (both the fully mature and high-mannose forms) remained stable for 24 h, whereas expression of all mutants declined within 2 h, and continued to decline over 24 h (Fig. 3). Thus, the fate of the nephrin mutants is consistent with ERAD. Interestingly, the S724C and R743C mutants, which were reported to be expressed in the plasma membrane (Liu et al. 2001), were degraded substantially, suggesting that only a small fraction escapes ERAD.

**Figure 4:**
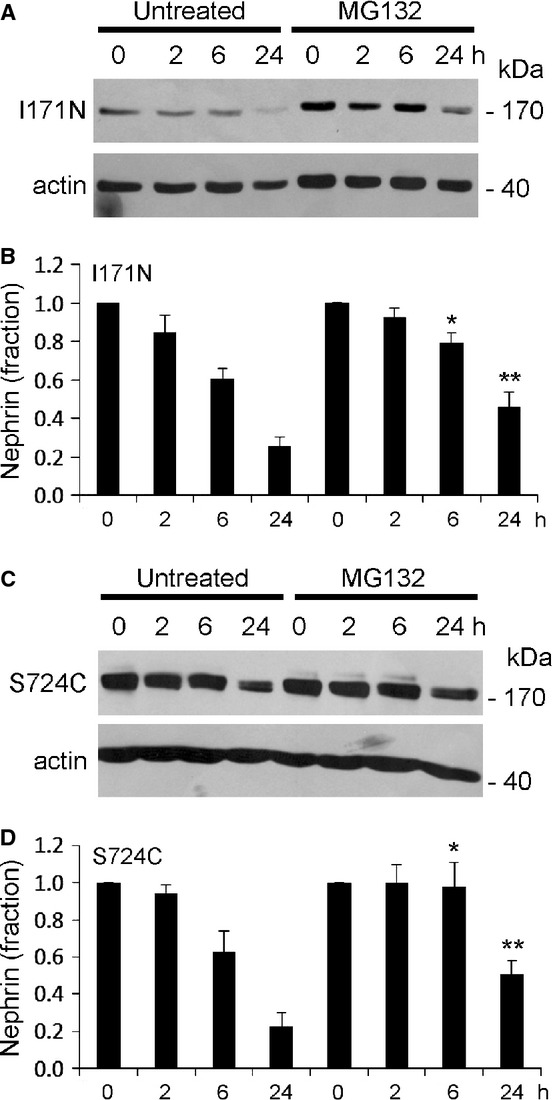
Proteasome inhibition reduces degradation of nephrin mutants. 293T cells were transfected with I171N and S724C nephrin mutants. Cells were treated with cycloheximide (as in Fig. 3), and with or without the proteasome inhibitor, MG132 (25 μg/mL). Representative immunoblots (A and C) and densitometric quantification (B and D) are presented. The degradation of nephrin was reduced by MG132. B: *P < 0.025 (MG132 vs. untreated at 6 h), **P < 0.035 (24 h), *N* = 4. (D) *P < 0.045 (MG132 vs. untreated at 6 h), **P < 0.015 (24 h), *N* = 4.

In a second set of experiments, we examined if inhibition of the proteasome would block degradation of nephrin. 293T cells were transfected with I171N and S724C nephrin mutants. Cells were treated with cycloheximide, and with or without the proteasome inhibitor, MG132 (at a dose that was not independently toxic to the cells). The degradation of nephrin at serial time intervals was reduced significantly by MG132, in keeping with ERAD (Fig. 4). It should be noted that degradation of nephrin mutants was not abolished completely by MG132. This drug most likely did not block proteasomal function completely, as complete proteasomal blockade is toxic to cells. Alternatively, other degradation pathways may be recruited if the proteasome is inhibited.

### Nephrin mutants undergo enhanced ubiquitination, but do not impair the function of the ubiquitin-proteasome system

**Figure 5:**
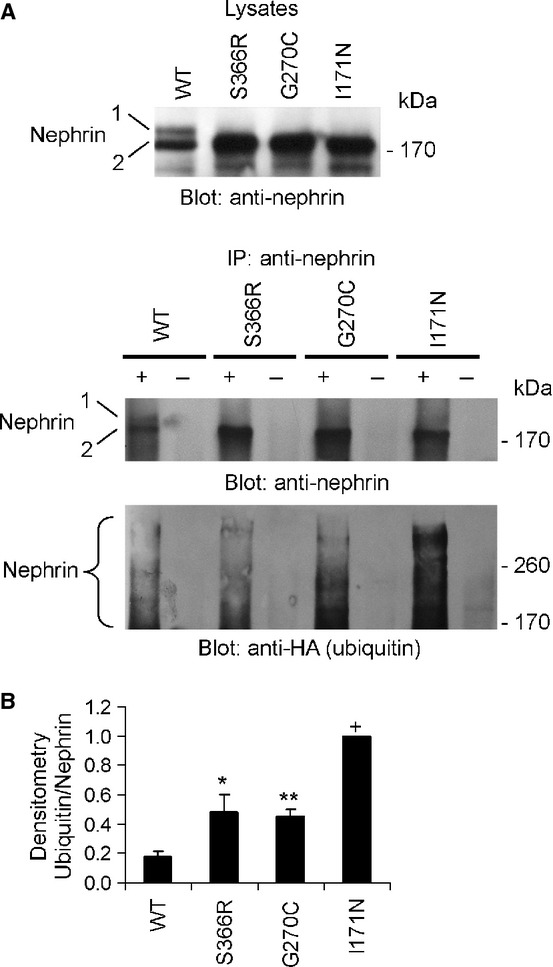
Ubiquitination of nephrin mutants. 293T cells were transfected with nephrin WT or mutants. In some experiments, cells were also transfected with HA ubiquitin. MG132 (25 μg/mL) was added 30 h after transfection (to block degradation of ubiquitinated proteins), and cells were harvested after 48 h. Lysates were immunoprecipitated with antibody to nephrin (+), or nonimmune IgG in controls (−). Immune complexes were immunoblotted with anti-HA or antiubiquitin antibody, as well as antinephrin antibody. (A) Representative anti-HA and antinephrin antibody immunoblots. (B) Densitometric quantification. As there were no significant differences in ubiquitination with HA-tagged (*N* = 3) versus endogenous ubiquitin (*N* = 3), the data were analyzed together. Compared with nephrin WT, the nephrin mutants showed increased ubiquitination. *P < 0.02, **P = 0.03, ^+^*P* < 0.0001 versus WT.

As noted above, the 26S proteasome is responsible for quality control by eliminating defective proteins from the cytosol and ER. Targeting of substrates to the 26S proteasome requires their prior tagging by covalently linked polyubiquitin chains (Navon and Ciechanover 2009; Dantuma and Lindsten 2010). As the nephrin mutants demonstrated enhanced degradation (Fig. 3), we examined if the three more severely misfolded mutants would also demonstrate enhanced ubiquitination prior to proteasomal degradation. In some experiments, a cDNA encoding for hemagglutinin antigen epitope (HA)-tagged ubiquitin was transiently transfected along with the S366R, G270C, and I171N nephrin mutants, whereas in other experiments, we monitored ubiquitination with endogenous ubiquitin. Cells were treated with MG132 to block proteasomal degradation of ubiquitinated proteins. Then, lysates were immunoprecipitated with antibody to nephrin, which was followed by immunoblotting of immune complexes with anti-HA or antiubiquitin antibodies (Fig. 5). The three nephrin mutants, in particular I171N, showed significantly increased ubiquitination, compared with WT nephrin.

**Figure 6:**
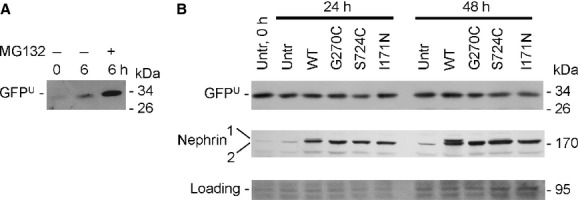
Effect of nephrin mutants on ubiquitin-proteasome system activity, as monitored by the GFP^U^ reporter. (A) 293 cells stably expressing GFP^U^ were incubated with (+) or without (−) MG132 for 6 h to block the proteasome. Lysates were immunoblotted with antibody to GFP. MG132 increased the level of GFP^U^. (B) 293 cells stably expressing GFP^U^ were transiently transfected with nephrin WT or mutants. Lysates were immunoblotted with antibodies to GFP or nephrin after 24 or 48 h. No differences in GFP^U^ were found between nephrin WT and mutants, implying that the mutants did not affect the global function of the ubiquitin-proteasome system. The immunoblot is representative of four experiments. Untr, untransfected. The faint band at ∼180 kDa in untransfected cells in the nephrin immunoblot is nonspecific. The GFP^U^ blot in panel A is underexposed, compared with the GFP^U^ blot in panel B. (Loading – Ponceau stain.)

Intracellular accumulation of excess misfolded and ubiquitinated proteins may be toxic to the function of the ubiquitin-proteasome system, and lead to its impairment (Menendez-Benito et al. 2005; Dantuma and Lindsten 2010). To investigate if misfolded nephrin proteins perturb the ubiquitin-proteasome system, we employed a clonal line of 293 cells, which stably expresses the ubiquitin-proteasome system reporter, GFP^U^. The GFP^U^ reporter consists of a short degron, CL1, fused to the C-terminus of GFP (Kitzler et al. 2012; Bence et al. 2001). A decline in the level of GFP^U^ reflects ubiquitin-proteasome system activity, whereas an increase indicates that the activity is reduced or impaired. As expected, incubation of the 293 cells with the proteasomal inhibitor, MG132, followed by immunoblotting of cell lysates with anti-GFP antibody resulted in an increased level of GFP^U^ (Fig. 6A), confirming that the reporter undergoes degradation only when the proteasome is active. The 293 cells were then transiently transfected with nephrin WT or nephrin mutants, and lysates were subjected to immunoblotting. There were no significant differences in GFP^U^ between nephrin WT and mutants at 24 and 48 h after transfection (Fig. 6B; four experiments, densitometry data not shown), indicating that the mutants did not affect global ubiquitin-proteasome system function.

### Nephrin mutants activate the UPR

**Figure 7:**
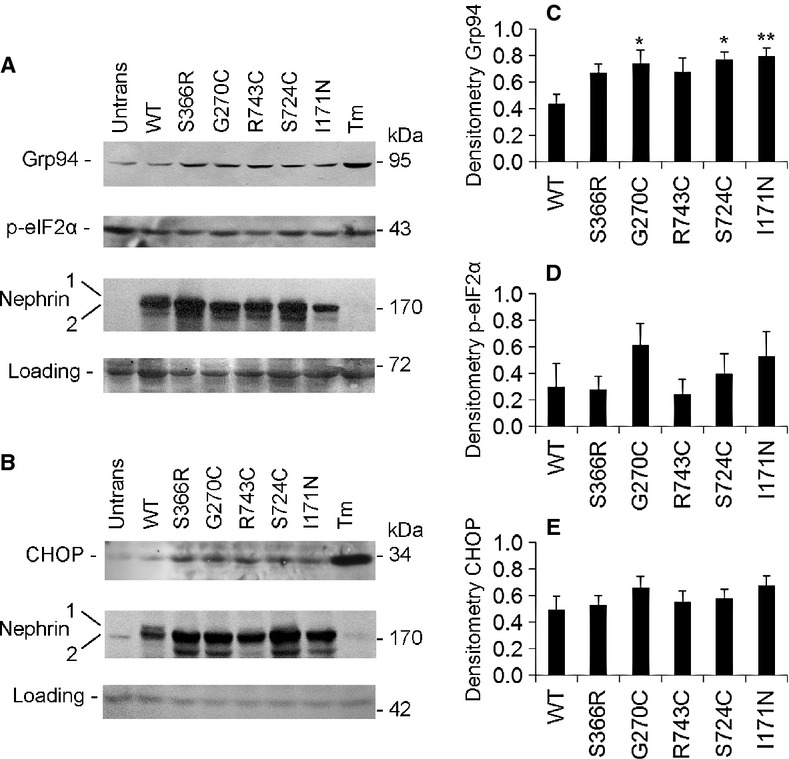
Nephrin mutants enhance Grp94 expression. 293T cells were transfected with nephrin WT or mutants. After 48 h, lysates were immunoblotted with antibodies to Grp94 (A), phospho (p)-eIF2α (A), and CHOP (B). A and B are representative immunoblots; C–E are densitometric quantifications. A significant increase in the expression of Grp94 was induced by three of five nephrin mutants. (C) *P < 0.05,**P < 0.005 versus WT, *N* = 4. Grp94 in untransfected (Untrans) cells, and the effect of tunicamycin (Tm; 10 μg/mL, 24 h incubation) on Grp94 are shown for comparison (A). There were no significant changes in the levels of eIF2α phosphorylation (A and D, *N* = 4) or CHOP expression (B and E, *N* = 12) induced by the nephrin mutants, compared with WT. There were no significant differences among the expression levels of nephrin WT and mutants in these experiments (A–E), as quantified by densitometry. (Loading – Ponceau stain.)

Accumulation of misfolded proteins in the ER can activate the UPR. To determine if misfolding of nephrin would activate the UPR, 293T cell was transiently transfected with nephrin WT and mutants, and expression of the ER chaperone, Grp94, was assessed by immunoblotting (Fig. 7A and C). The I171N, G270C, and S724C mutants increased expression of Grp94. The S366R mutant tended to increase Grp94, but the changes were not consistent among all experiments, and the R743C mutant did not enhance Grp94 expression significantly. It should be noted that transfection of cells with nephrin WT did not affect the basal level of Grp94 expression (basal level in untransfected cells 0.46 ± 0.11 units vs. nephrin WT transfection 0.44 ± 0.07 units, shown in Fig. 7A and C). Thus, transfection or expression of an ectopic protein that is targeted to the ER did not induce Grp94.

To address whether nephrin mutants activate the PERK pathway, eIF2α phosphorylation was monitored by immunoblotting with a phospho-specific antibody (Fig. 7A and D). The nephrin mutants did not increase eIF2α phosphorylation significantly. Phosphorylation of eIF2α can also upregulate the expression of ATF4, leading to enhanced transcription of CHOP. In keeping with the lack of effect on eIF2α phosphorylation, the nephrin mutants had no consistent effect on CHOP expression (Fig. 7B and E).

**Figure 8:**
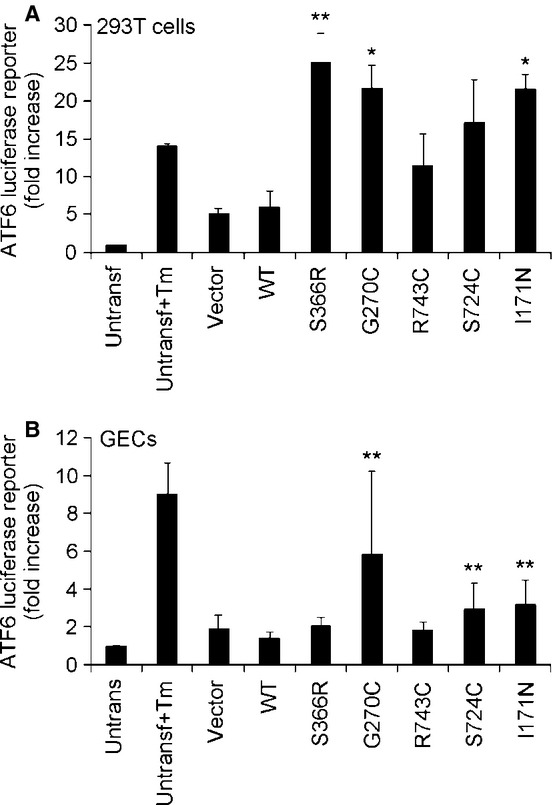
Nephrin mutants enhance ATF6-luciferase reporter activity. 293T cells (A) or GECs (B) were cotransfected with nephrin WT or nephrin mutants, an ATF6 firefly luciferase reporter plasmid, and renilla luciferase (marker of transfection efficiency). Lysates were assayed for luciferase activity after 48 h. Normalized luciferase activity is presented in arbitrary units. A significant increase in ATF6 luciferase activity was induced by several nephrin mutants. (A) *P < 0.05,**P < 0.005 versus WT, *N* = 3; B: **P < 0.005 versus WT, *N* = 5. The effect of tunicamycin (Tm) in untransfected (Untransf) cells and empty vector transfections are presented for comparison.

The increase in Grp94 induced by several nephrin mutants was consistent with activation of the ATF6 or inositol-requiring-1α pathways. To demonstrate activation of ATF6, we employed a cDNA reporter construct containing five tandem copies of an ATF6 response element fused to firefly luciferase (p5xATF6-GL3) (Wang et al. 2000). 293T cells were cotransfected with nephrin WT or mutants, and the ATF6 luciferase reporter. A significant increase in ATF6 reporter activity was induced by three nephrin mutants, including S366R, G270C, and I171N (Fig. 8A). An analogous experiment was performed in GECs, and three nephrin mutants increased ATF6 luciferase activity, including I171N, G270C, and S724C (Fig. 8B). Stimulation was lower in GECs, most likely due to low transfection efficiency. In summary, the I171N and G270C nephrin mutants (two of the most severely misfolded mutants) significantly enhanced expression of Grp94, and ATF6 reporter activity in the two cell lines. The S366R and S724C mutants induced significant increases in these parameters in some of the assays, whereas the R743C mutant consistently showed no significant effect. The nephrin mutants did not stimulate phosphorylation of eIF2α, nor induction of CHOP. Together, the results support selective activation of the UPR, in particular the ATF6 branch, which is regarded as an adaptive/cytoprotective pathway. We cannot exclude activation of the inositol-requiring-1α pathway by nephrin mutants, but PERK was not activated.

### Castanospermine enhances expression of nephrin mutants in the plasma membrane

To restore the function of nephrin in maintaining glomerular permselectivity, the initial step would be to enhance expression of mutant nephrin molecules in the podocyte plasma membrane. In the next series of experiments, we addressed whether export of nephrin mutants to the plasma membrane could be enhanced, thereby improving expression and function. Sodium 4-phenylbutyrate was previously shown to function as a “chemical chaperone,” that is, a drug that can assist with the folding of certain misfolded or mutant proteins in the ER and correct cellular trafficking of these proteins to the plasma membrane (Araki and Nagata 2011; Guerriero and Brodsky 2012). In an earlier study, sodium 4-phenylbutyrate was effective in rescuing the export of certain nephrin mutants, including S366R, but was not effective with others, including I171N (Liu et al. 2004). We tested if plasma membrane expression of the S366R and I171N nephrin mutants can be “rescued” by modulating their interactions with an ER chaperone, and whether this approach could be more effective than sodium 4-phenylbutyrate. The interaction of nephrin with calnexin facilitates nephrin folding, but excessive interactions potentially lead to ERAD. Given that nephrin mutants were associated with calnexin (Fig. 1), reducing the interaction of a misfolded nephrin with calnexin could potentially facilitate its export to the plasma membrane (Patterson and Reithmeier 2010). Castanospermine, an inhibitor of glucosidase I, blocks glucose trimming and disrupts interactions of calnexin with substrates (Patterson and Reithmeier 2010). Immunostaining with the antibody to the nephrin cytoplasmic domain showed that treatment of 293T cells with castanospermine did not change the expression of nephrin WT in the plasma membrane (Fig. 2G); however, castanospermine increased the amount of nephrin S366R and I171N appearing in the plasma membrane (Fig. 2H and I). In all cases, nephrin was also present in the ER (Fig. 2G–I). Using the antibody directed to the nephrin extracellular domain, we showed that castanospermine increased cell surface staining (Fig. 2N and O). The results suggest that the drug indeed reduced ERAD and allowed export of these nephrin mutants to the plasma membrane. Quantification of nephrin plasma membrane expression was carried out by measuring the total fluorescence intensity of cell surface nephrin staining and normalizing by the intensity of F-actin staining, which reflects cell number. Castanospermine induced an almost twofold increase in cell surface expression of nephrin S366R and I171N (Fig. 2P). Although significant, the rescue of mutant nephrin expression with castanospermine was partial, in the range 30–35% of plasma membrane expression of nephrin WT (Fig. 2P).

**Figure 9:**
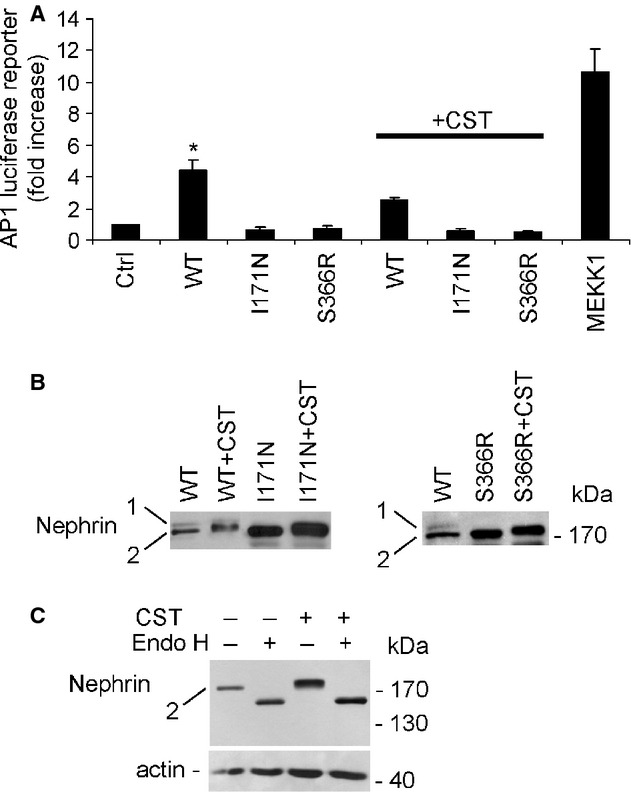
Effect of castanospermine on nephrin signaling and glycosylation. 293T cells were transfected with nephrin WT, or the S366R and I171N mutants. In panel A, cells were cotransfected with an AP1 firefly luciferase reporter plasmid and renilla luciferase. Some cells were incubated with castanospermine (CST; 1 mmol/L, 18 h). (A) Lysates were assayed for luciferase activity after 48 h. Normalized luciferase activity is presented in arbitrary units. The nephrin mutants were unable to stimulate AP1 luciferase activity, compared with nephrin WT. Castanospermine did not enhance AP1 luciferase activity of the nephrin mutants. The effect of constitutively active MEKK1 transfection on AP1 luciferase activity is shown for comparison. *P < 0.0005 WT versus empty vector control (Ctrl), *P* < 0.0001 WT versus I171N, *P* < 0.0001 WT versus S366R, *N* = 3–6. (B and C) Cell lysates were immunoblotted with antinephrin antibody. Castanospermine induced a slight upward mobility shift in the partially glycosylated forms of nephrin WT and mutants (band 2). (C) Endoglycosidase H (Endo H) induced a complete loss of nephrin I171N (band 2) and the appearance of faster migrating bands (nonglycosylated nephrin) in both untreated and castanospermine-treated cells.

In vivo, a reduction in proteinuria would imply that the rescued nephrin mutant is functionally effective, but measurement of proteinuria is not possible in cell culture. However, nephrin also has the capability of transducing signals in cells (Huber et al. 2001; Patrakka and Tryggvason 2007; Chuang and He 2009; Hattori et al. 2011). As proof of principle for nephrin signaling, we monitored the ability of nephrin to induce activation of an AP1-luciferase reporter in 293T cells (Huber et al. 2001; Quack et al. 2006), which was shown to occur via the p38 and c-Jun N-terminal kinase pathways (Huber et al. 2001). In untreated cells, nephrin WT, but not the S366R and I171N mutants, induced robust activation of the AP1 reporter (Fig. 9A). This result implies that expression of nephrin at the plasma membrane is required for signaling to AP1. Treatment of cells with castanospermine did not, however, increase the activation of the AP1 reporter by the S366R and I171N nephrin mutants (Fig. 9A). Therefore, even though castanospermine enhanced the expression of the nephrin mutants in the plasma membrane, these mutants were unable to enhance AP1 signaling.

Finally, we examined the effect of castanospermine on nephrin glycosylation, by monitoring migration with SDS-PAGE. The major effect of castanospermine was to induce an upward gel-mobility shift in the immature, high-mannose form of nephrin, that is, the principal form observed in the S366R and I171N mutants (Fig. 9B and C, band 2). This effect was probably due to additional glucose molecules being bound to the high-mannose structures at multiple N-glycosylation sites following inhibition of glycosidase I. However, treatment with castanospermine did not appear to result in the conversion of the S366R and I171N nephrin mutants into the fully mature form of nephrin, observed in nephrin WT (Fig. 9B, band 1). Castanospermine also induced an upward gel shift in the high-mannose form of nephrin in the WT. The fully mature form also appeared to shift upward, although it was difficult to resolve the fully mature from the high-mannose form by SDS-PAGE, given the upward shift in the latter (Fig. 9B). Incubation of lysates of cells expressing I171N nephrin with endoglycosidase H resulted in a complete loss of the nephrin bands and the appearance of faster migrating bands (nonglycosylated nephrin) in both untreated and castanospermine-treated cells (Fig. 9C). This experiment confirms that castanospermine modified high-mannose structures in the nephrin mutant, but did not result in full maturation, that is, addition of complex oligosaccharides.

## Discussion

This study demonstrates that certain human disease–associated mutants of nephrin accumulate in the ER, show impaired maturation and enhanced association with calnexin, undergo ubiquitination and ERAD, and lead to the induction of ER stress (UPR). Furthermore, severely misfolded nephrin mutants were poorly expressed in the plasma membrane, and reducing the interaction of these mutants with calnexin enhanced plasma membrane expression significantly. Full maturation and AP1 signaling activity of these mutants were, however, not restored. By immunofluorescence microscopy, we demonstrated that nephrin WT was localized in the plasma membrane, although there was also some localization in the ER (Fig. 2). The S366R, I171N, and G270C nephrin mutants primarily localized in the perinuclear region, together with the ER marker calnexin (Fig. 2), in keeping with earlier studies (Liu et al. 2001; Barletta et al., 2003). These patterns of localization were consistent with the posttranslational forms of nephrin, that is, the mutants (found in the ER) were expressed only as immature, high-mannose forms, whereas the WT (in the ER and plasma membrane) was expressed as both high-mannose and complex oligosaccharide forms (Fig. 1). In cultured cells, the high-mannose form was expressed at levels comparable or higher than the fully mature form, while expression of the mature form was greater in the glomerulus. More efficient nephrin maturation in the glomerulus, compared with cultured cells, may have accounted for these differences. Alternatively, the high-mannose form may have been degraded more rapidly in vivo, compared with cultured cells. The cultured GECs in this study did not express endogenous nephrin, consistent with various GEC lines; nevertheless, other GEC lines express endogenous nephrin, and by analogy to ectopic nephrin, the localization of endogenous nephrin was both in the plasma membrane and perinuclear region (Saleem et al. 2002; Schiwek et al. 2004).

Interactions between newly synthesized glycoproteins and ER chaperones provide a quality control mechanism by which glycosylated and correctly folded proteins are exported to the Golgi, and subsequently to the plasma membrane, or are secreted. Underglycosylated and incorrectly folded glycoproteins may undergo ERAD (Parodi 2000). Calnexin recognizes monoglucosylated glycoproteins (Deprez et al. 2005; Caramelo and Parodi 2008), and as nephrin contains up to 10 potential N-glycosylation sites (Khoshnoodi et al. 2007), nephrin would be predicted to interact with calnexin while undergoing maturation in the ER. In this study, nephrin mutants showed conspicuous association with calnexin (Fig. 1). Most likely, this reflects impaired folding of the mutants during maturation, resulting in their prolonged association with calnexin while attempting to fold correctly. By analogy, a previous study had shown that glucose starvation of cells led to formation of underglycosylated nephrin that remained in the ER as a complex with calnexin and calreticulin (Fujii et al. 2006).

A fraction of proteins passing through the ER will remain terminally misfolded, and removal of these polypeptides via ERAD is essential to ensure proper cell function (Hirsch et al. 2009; Araki and Nagata 2011; Bernasconi and Molinari 2011; Guerriero and Brodsky 2012). Under resting conditions, ERAD capacity is generally sufficient to dispose misfolded proteins, whereas induction of the UPR can upregulate certain ERAD components. Nephrin mutants underwent a time-dependent proteasomal degradation, consistent with ERAD, while expression of nephrin WT was stable in cells for at least 24 h (Fig. 3). Moreover, the S366R, I171N, and G270C mutants showed increased ubiquitination, compared with WT nephrin (Fig. 5), implying that a significant proportion of the mutant proteins were targeted for proteasomal degradation.

It has been reported that an overabundance of misfolded or damaged proteins, including proteins that transit through the ER, for example, a mutant cystic fibrosis transmembrane conductance regulator (CFTR), can form potentially toxic aggregates that could impair the function of the ubiquitin-proteasome system (Bence et al. 2001). To investigate the specific relation between misfolded nephrin proteins and ubiquitin-proteasome function, we employed 293 cells stably expressing the proteasomal reporter, GFP^U^ (Kitzler et al. 2012; Bence et al. 2001). After transfection of nephrin, no apparent differences in the levels of GFP^U^ were found between WT and mutants (Fig. 4), suggesting that misfolding of nephrin mutants did not impair global ubiquitin-proteasome function. This result may be related to lack of protein aggregate formation by the nephrin mutants.

Mutant nephrin misfolding in the ER nevertheless induced the UPR (Ron and Walter 2007; Zhang and Kaufman 2008; Hetz 2012). The more severely misfolded mutants, which are largely retained in the ER (I171N and G270C), enhanced Grp94 expression in 293T cells, and ATF6-luciferase activity in both 293T cells and GECs (Fig. 7 and 8). The R743C mutant (which has been detected in the plasma membrane, and is probably less misfolded, compared with the former) did not enhance Grp94, nor ATF6 activity. S366R and S724C stimulated the UPR in some assays; the discrepancies in their effects may have been related to sensitivities of the assays, or different processing in distinct cell lines. The ATF6 branch of the UPR is generally considered to be adaptive or cytoprotective, implying that the role of the UPR triggered by the mutant nephrin is to confer protection to the cells from any potential proteotoxic effects of misfolded proteins. The PERK–ATF4–CHOP pathway, which mediates ER stress-induced apoptosis (Yoshida 2007; Hetz 2012), was not activated by any of the nephrin mutants, suggesting that the ER stress was not of sufficient intensity to result in apoptosis. In addition to nephrin, the effects of other mutant glycoproteins on the induction of ER stress have been examined previously. For example, a mutant low-density lipoprotein receptor was able to induce ER stress (Sorensen et al. 2006). In the case of mutant CFTR, induction of ER stress has been reported in some, but not all studies (Xu et al. 2006; Rab et al. 2007). Conversely, various forms of renal injury are reported to be associated with the induction of ER stress (Cybulsky 2010). In particular, ER stress has been demonstrated in podocyte injury and proteinuria, and may be related to excessive global misfolding of proteins in the ER. ER stress under conditions of glucose deprivation, or in experimental glomerular injury, may be associated with impaired nephrin folding, and amelioration of ER stress in glomerular injury can lead to an improvement in nephrin expression in the plasma membrane (Fujii et al. 2006; Nakajo et al. 2007).

Studies on misfolded mutant proteins have shown that ER quality control systems can be manipulated (Araki and Nagata 2011; Guerriero and Brodsky 2012). The chemical chaperone, sodium 4-phenylbutyrate, was able to facilitate plasma membrane expression of some, but not all, nephrin mutants (Liu et al. 2004). Another approach that was shown to increase plasma membrane expression of mutant kidney anion exchanger proteins employed castanospermine to reduce the interaction of these misfolded proteins with calnexin, thereby reducing ERAD and facilitating export to the plasma membrane (Patterson and Reithmeier 2010). We showed that castanospermine significantly increased the amount of nephrin I171N and S366R in the plasma membrane (Fig. 2), in keeping with a partial “rescue” of the mutant phenotype. The amounts of rescued nephrin mutants measured on the cell surface (Fig. 2) are in keeping with the effect of castanospermine on the rescue of kidney anion exchanger mutants (Patterson and Reithmeier 2010), as well as with the rescue of CFTR by several individual chemical compounds (Okiyoneda and Lukacs 2012). Even a relatively low-level rescue of nephrin mutants could potentially enhance the homotypic interactions of nephrin and assembly of filtration slit diaphragms, and reduces proteinuria in vivo. In contrast to nephrin and the kidney anion exchanger proteins, castanospermine was apparently not effective in rescuing plasma membrane expression of a mutant CFTR (Chang et al. 2008).

Unexpectedly, the castanospermine-rescued I171N and S366R nephrin mutants in the plasma membrane were not able to transduce signals, as monitored by an AP1-luciferase reporter assay (Huber et al. 2001) (Fig. 9). In contrast, it had been reported that nephrin mutants rescued with sodium 4-phenylbutyrate (including S366R) were able to undergo tyrosine phosphorylation after antibody-induced cross-linking of the extracellular domain of nephrin (Liu et al. 2004). We were not able to employ the latter assay, as the antibody to the nephrin extracellular domain used in this study could not induce tyrosine phosphorylation in WT nephrin. The signaling aspects of nephrin may play a role in maintaining proper interactions with certain adaptors and cytoskeletal proteins in the podocyte (Patrakka and Tryggvason 2007; Chuang and He 2009; Hattori et al. 2011), but the extent to which defects in nephrin signaling pathways compromise the structure of the slit diaphragm and maintenance of glomerular permselectivity is an evolving area that requires further study. Whether castanospermine was able to restore the function of the rescued mutant kidney anion exchanger was not assessed in the earlier study (Patterson and Reithmeier 2010).

Further analysis of the castanospermine effect on the nephrin mutants suggested that by blocking glycosidase I, the drug increased the amount of glucose molecules in the high-mannose, immature form of nephrin, thereby inducing a gel-mobility shift (Fig. 9). Castanospermine did not, however, facilitate conversion of the immature form of nephrin into the fully mature form (Fig. 9). Possibly, additional glucosylation of the immature form impaired addition of complex oligosaccharide or other posttranslational modifications from occurring in the Golgi. Thus, even though plasma membrane expression of the high-mannose forms of nephrin was enhanced, failure to achieve conversion to the fully mature form may have prevented nephrin from activating AP1 effectively. These results also raise the possibility that castanospermine facilitated delivery of the high-mannose forms of the I171N and S366R nephrin mutants from the ER to the plasma membrane by an unconventional secretion pathway (Gee et al. 2011). This postulate will require further study. Whether fully mature glycosylation of nephrin S366R occurred after sodium 4-phenylbutyrate treatment was not established in the earlier study (Liu et al. 2004).

In conclusion, nephrin missense mutants, which are misfolded in the ER, are ubiquitinated, undergo ERAD, and trigger an adaptive UPR. Reducing the interaction of nephrin mutants with calnexin using small molecules, perhaps in combination with chemical chaperones and regulators of proteostasis, can be employed to increase plasma membrane expression. Further studies will be required to determine how effectively the rescue of nephrin mutants reduces proteinuria. Novel approaches to the modulation of ER quality control may evolve into effective therapies for proteinuric kidney disease, including congenital nephrotic syndrome.
